# COVID 2019 pandemic: a true digital revolution and birth of a new educational era, or an ephemeral phenomenon?

**DOI:** 10.1080/10872981.2020.1781378

**Published:** 2020-06-18

**Authors:** Yassamine Bentata

**Affiliations:** Nephrology and Kidney Transplantation Unit, Mohammed VI University Hospital, Oujda, Mohammed First University, Oujda, Morocco; Laboratory of Epidemiology, Clinical Research and Public Health, Medical School, Mohammed First University, Oujda, Morocco

## Personal view

The COVID-19 pandemic is a true apprenticeship; it has obliged the whole world to use technological tools to ensure teleworking and distance learning. In record time, it has imposed education completely by distance on millions of students and has forced institutions and countries to invest in and fully adapt to it very rapidly. Great financial means has been injected and colossal efforts have been made by all the countries to succeed in the enormous project and save the current academic year. The subject deserves serious reflection and an analysis of the strong and weak points in these unique circumstances, never before experienced.

The COVID-19 pandemic began unpredictably and abruptly in December 2019 in Wuhan, China and quickly spread to more than 200 countries on five continents. The world has been accustomed to living through and overcoming major viral pandemics like the H5N1 *(‘*Avian flu*’*) in 2004, H1N1 in 2009 and Ebola in 2012 of this Century. These pandemics have caused great human and economic losses, but the COVID-19 pandemic is the world’s greatest medical catastrophe, having also caused the most economic losses since the Second World War. This pandemic has come at a time when countries felt untouchable, individuals felt invulnerable, and that their economic power and technological development protected them from all danger. Any threats feared up to then were mainly predictable, of the economic, military and/or political type, while microbiological threats, particularly viral, had never appeared as a real danger in this twenty-first century. While the COVID-19 pandemic is a real human and economic catastrophe, it has allowed citizens and leaders to become aware of the failings of our systems, failings newly identified and up to now ignored.

The confinement imposed by this pandemic, which has involved more than half of the world’s inhabitants, right in the middle of an academic year, has required States to continue providing education by offering students distance learning, a form of education that is meant to be pedagogically sound, of high quality and effective. Distance learning has thus become one of the great challenges of this pandemic, since most of the countries concerned were not prepared for such an unprecedented situation. In Morocco, we knew that our medical education system is not very robust, has recently undergone a major revision of pedagogical objectives and pedagogical teaching programs and a reform of the overall architecture of medical studies including medical specialty training is underway. This crisis would reveal all its main weaknesses and difficulties and put all of its components to the test. The current context is also a good opportunity to improve these identified weaknesses.

Confinement measures in our country began on March 16, and continue as of today, 20 May 2020, and since the start of confinement, courses have been conducted remotely. Neither the students, nor the teachers, nor the institutions, were prepared for such a situation. With regard to medical studies, all courses were suspended: lecture courses, tutorials, lab work and hospital rotations for the first 5 years of medical studies. For the sixth-year hospital internships, the students practiced either in a high-activity area of COVID-19 patients, or in a low-activity area of non-COVID-19 patients, but in both cases, it was impossible to achieve the institutional, general, and specific objectives of the hospital internships.

For the first 5 years of medical studies, courses were put online using the e-learning platform of our university, University Mohammed First of Oujda, Morocco, a platform that is quite well developed and diversified. These courses were accompanied by audio sequences by the teachers with explanations and detailed course descriptions. Some teachers posted videos recorded in the auditoriums, without students during confinement, particularly for teaching of anatomy using the chalkboard for instructional diagrams with comments. Other teachers organized online courses for large groups of students using modern communication tools (zoom.us, google meeting, google classroom, Facebook, etc.) and/or review and discussion sessions to interact with the students and answer their questions. Hundreds of entire class sessions, of 2 h each, had been recorded in the 2 years preceding confinement, that are accessible to students and constitute a good base of support. This distance learning is a good alternative for theoretical instruction, usually conducted in the Faculty auditoriums, with the main objective of ensuring acquisition of a solid knowledge of fundamental, preclinical, and clinical sciences, and falling mainly in the realm of core knowledge. Practical teaching, in contrast, is usually conducted during rotations at the patient’s bedside, within the departments of the university hospital, and the main objective is to develop the medical skills to resolve simple, but especially complex clinical situations. Distance learning as it is conducted for core knowledge cannot meet this need. More active methods, such as online organization of practice sessions in clinical reasoning skills, discussion of clinical cases, role playing, simulated scenarios, participation in script concordance tests (SCT), portfolio writing, or other active methods allowing the development of these skills, should be implemented, but demand good mastery of these techniques, strong motivation and an adequate number of teachers and senior advisers. Medical simulation, a very powerful and effective tool for skills development should be integrated into this and distance learning. Medical simulation software, similar to video games, in different fields of medicine and including hundreds of scenarios, should be developed with the remote participation of numerous students, both student ‘actors’ and student ‘observers’.

Distance learning, under the conditions of the pandemic-dictated confinement, pushes us to reflection and convinces us of its importance; even those teachers who had been the most reticent about new technologies have been very supportive and strongly involved. On the other hand, the new generations of students are familiar with these digital tools that they particularly appreciate and enjoy using, and thus adapted well and rapidly. However, for some students, this situation was stressful and a source of unease and confusion because the digital tool is synonymous with social media, relaxation, chat, new encounters, and to see this tool suddenly turn from a ‘cool’ distraction into one of hard work and serious concentration, may be distressing for some. As for the institutions, they lacked the human and technical resources necessary to fully carry out this mission of online courses. It should be pointed out that online teaching for a class of 30 students in an engineering school calls for relatively simple organization and maintains an environment propitious for learning. In contrast, online teaching for a class of 400 students in a medical school, with the obligation to develop their skills, is very difficult to set up and alters the learning environment that remains a major condition for university medical training. This may lead to total loss of motivation of the students and disinterest of the faculty, with failure and abandonment of studies by students, especially during the first 2 years of medical studies, when students are the most vulnerable because they are very young, a bit lost in their career choice, and not yet having attained the maturity and resistance of older adults. We approached the teaching issue only in its “transmission of knowledge“ aspect. Assessments were not attempted, as most countries announced the return to school and the new academic year for September, and postponed the period of assessment to September, to have time to discuss and plan. Although distance learning has proved to be quite efficient and effective, many difficulties and uncertainties arise about the feasibility of distance assessment.

Whatever the conditions, supportive measures are necessary, on the individual (teacher and student), institutional and national levels. Adequate training is needed for educators in technological, pedagogical, and IT tools enabling them to be more skilled and independent in using the different e-learning platforms, and creative and innovative in their teaching methods. In addition, reliable internet access and availability of individual computers for all students are required, as well as the recruitment of qualified technical personnel to develop and maintain the e-learning platform with better security, confidentiality, and effectiveness.

We must also applaud the outstanding efforts made by all those in charge and all actors concerned for the implementation of distance learning, a successful experiment that saved the academic year for thousands of Moroccan students. Weak points have been identified and will be gradually improved until a satisfactory quality of distance learning is achieved.

Will the COVID-19 pandemic see the birth of a true digital revolution and the start of a new era of distance learning, or will we revert to our former learning environment, classical and not very modern, of the time before confinement? Will our students prefer to return to the agitated benches of the auditoriums or stay comfortably at home and follow courses online? We will have clear answers when life returns to normal, but one thing is certain, that the future investment of humanity is in digital technology, which will be an extraordinary competitive market to develop.

